# *Culicoides insignis* in Ecuador: Molecular identification of blood meals and detection of bluetongue virus

**DOI:** 10.1016/j.crpvbd.2025.100288

**Published:** 2025-06-25

**Authors:** Juan D. Mosquera, Sonia Zapata, Denis Augot

**Affiliations:** a Instituto de Microbiología, Colegio de Ciencias Biológicas y Ambientales COCIBA, Universidad San Francisco de Quito (USFQ), Quito, Ecuador; b USC VECPAR, ANSES-LSA-URCA, Faculté de Pharmacie Université de Reims Champagne-Ardenne Reims, EA7510, France; c ANSES, INRAE, ENVA, UMR-BIPAR, Laboratoire de Santé Animale, 14 rue Pierre et Marie Curie, Maisons-Alfort Cedex, 94701, France

**Keywords:** Bluetongue virus, Blood meal, *Culicoides insignis*, Vector, Ecuador, Midges

## Abstract

Bluetongue virus (BTV) is an *Orbivirus* transmitted by *Culicoides* biting midges and is the causative agent of bluetongue disease in wild and domestic ruminants. *Culicoides insignis* is the primary vector of BTV in Florida, Central America, the Caribbean, and South America. In Ecuador, recent investigations have reported the presence of BTV in cattle and identified *C. insignis* as the dominant species in localities from the Amazon Basin and Andean foothills. Understanding the host-feeding patterns of blood-feeding insects and evaluating their role in pathogen transmission are essential for elucidating the epidemiology of vector-borne diseases. To that end, we investigated the presence of BTV in unengorged *C. insignis* females collected in two localities: Cotundo, where BTV had previously been reported in cattle, and Paraiso Escondido located near (∼35 km) a site with a known history of BTV circulation. A total of 1773 female *Culicoides* spp. specimens were collected, of which 326 (18.38 %) were *C. insignis*. We identified the blood meal sources of engorged *C. insignis* females from both localities. Our results provide additional evidence to support the role of *C. insignis* as vector of BTV in Ecuador.

## Highlights

• *Culicoides insignis* is abundant in Cotundo, an Ecuadorian locality with a history of BTV transmission.
• Molecular identification of blood meals confirmed that *C. insignis* feeds on horses and humans in the Ecuadorian Amazon.
• The RT-qPCR detection of BTV RNA in one unengorged *C. insignis* female suggests a high viral load.
• This is the first molecular evidence of BTV in *C. insignis* from Ecuador.
• *Culicoides insignis* could be a potential vector of BTV in Ecuador.

## Graphical abstract

**Figure:**
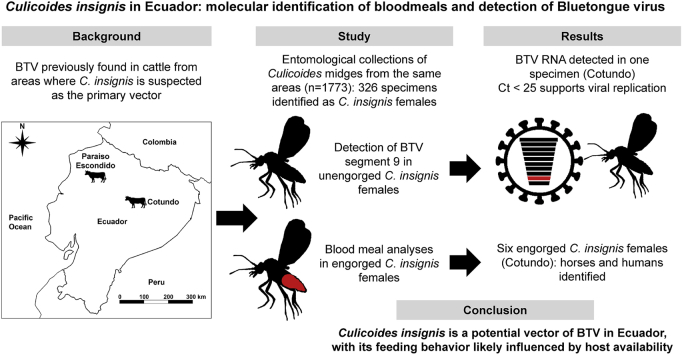

## 1 Introduction

Bluetongue virus (BTV) is an *Orbivirus* and the causative agent of bluetongue (BT) disease, a non-contagious infection that affects wild and domestic ruminants. The World Organization for Animal Health (WOAH; formerly OIE) classifies BT as a notifiable disease, given its considerable impact on livestock health and commerce. Global economic losses attributed to BT outbreaks are estimated at around US$3 billion annually due to factors such as mortality, decreased productivity, and trade restrictions (Rushton and Lyons, 2015).

The transmission of BTV between ruminants occurs primarily through *Culicoides* biting midges. Therefore, BTV distribution depends on the presence of *Culicoides* vector species in different ecosystems of temperate, subtropical, and tropical regions (Gibbs and Greiner, 1994; MacLachlan and Osburn, 2006).

Based on epidemiological data, the primary suspected *Culicoides* vectors of BTV are relatively well defined by region (Mellor et al., 2000): North America (*C. sonorensis* and *C. variipennis*); South America (*C. insignis*); Africa (*C. imicola*); Australia (*C. brevitarsis* and *C. wadai*); Asia and Indonesia (*C. fulvus*); and Europe (*C. imicola*). Other species have also been identified as additional vectors of BTV, such as *C. obsoletus* and the *Pulicaris* group (Tabachnick, 2004; Mehlhorn et al., 2007, 2009; Wilson and Mellor, 2009) in northern Europe and species of the subgenus *Avaritia* as well as *Schultzei* and *Milnei* groups in Africa (Hadj-Henni et al., 2023). Identifying specific vectors in each region is essential for developing effective control and prevention measures against BT (Caracappa et al., 2003).

In Ecuador, entomological surveys have revealed the presence of *C. insignis* across different regions, including the Amazon Basin, Andean foothills, and coastal areas (Mosquera et al., 2022). Additionally, alarming serological evidence indicates high occurrences of BTV (98.9%) and the Epizootic hemorrhagic disease virus (81.3%) in cattle and sheep from Ecuadorian slaughterhouses and dairy farms (Verdezoto et al., 2018). Despite this, no clinical cases of BT have been reported in Ecuador to date, and information regarding reservoirs and hosts remains scarce.

Understanding the host-feeding patterns of hematophagous insects such as *Culicoides* spp. is crucial to elucidate the epidemiology of vector-borne diseases. The host range of biting midges in South America remains largely undescribed. However, in the Neotropical region, *Culicoides* species are suspected to be mammalophilic or ornithophilic/mammalophilic (Blanton and Wirth, 1979; Carvalho et al., 2021; González et al., 2022).

Therefore, the first objective of the present study was to evaluate and quantify the presence of BTV in unengorged *C. insignis* females from Cotundo and Paraíso Escondido, using molecular methods, as a first step toward assessing the vectorial capacity of this species for BTV in Ecuador. The second objective was to assess the host associations of *C. insignis* by PCR amplification and sequencing of cytochrome *b* (*cyt*b) and prepronociceptin (PNOC) markers using blood-engorged females from the same localities.

## 2 Materials and methods

### 2.1 Entomological collections

**Fig. 1:**
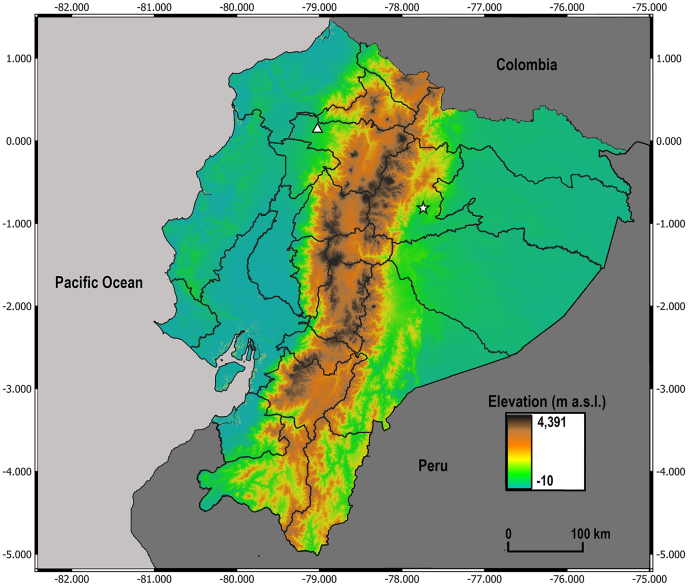
Map of Ecuador showing provincial divisions, the elevation gradient and the two localities where *Culicoides* were collected: Cotundo (*star*), located in Napo Province, and Paraíso Escondido (*triangle*), located in Pichincha Province.

CDC-like light traps were placed at different points in the localities of Paraiso Escondido (Andean foothills, Pichincha Province) and Cotundo (Amazon Basin, Napo Province) between February and April 2017 (Fig. 1). In Cotundo, BTV was previously confirmed in cattle through serological testing and virus isolation from blood samples, whereas Paraíso Escondido is located approximately 35 km from Tandapi (Santo Domingo Province), where BTV-positive cattle were also reported (Verdezoto et al., 2018). The traps were suspended about 1.5–2.0 m above the ground at the edge of secondary forests, near pastures, and avoiding areas close to other light sources, and sites exposed to strong winds. The traps were set at 18:00 h and collected the following morning at 6:00 h, to overlap with the times of major activity of midges. Collected specimens were sent to the Laboratory of Parasitology and Vectors of the Institute of Microbiology at the Universidad San Francisco de Quito, Quito, Ecuador, and preserved in 70% ethanol at −20 °C. All collections and analyses were carried out under the permit MAE-DNB-CM-2018-0085.

### 2.2 Morphological identification of *Culicoides* spp. and *Culicoides insignis*

Preliminary identification of individuals of the genus *Culicoides* was based on the morphological characteristics and wing patterns described in various identification keys (Forattini, 1957; Forattini et al., 1958; Wirth et al., 1988; Spinelli et al., 1993; Felippe-Bauer et al., 2003, 2008) using a stereomicroscope (Olympus SZ51). Specimens of *C. insignis* were identified based on their characteristic wing patterns (Spinelli et al., 1993) using a microscope (Leica 13595XXX) equipped with a digital camera (AmScope MU1803) after mounting of wings between slide and cover slide with Gum Chloral (Augot et al., 2017). After species identification, *C. insignis* females were separated as engorged (distended abdomen with red pigmentation) for blood meal analysis and unengorged (flat and pale abdomen) for BTV detection, based on Dyce (1969). Engorged and unengorged specimens were placed in 1.5-ml Eppendorf tubes individually and stored at −80 °C for nucleic acids extraction (Augot et al., 2010). All specimens were deposited in the collections of the San Francisco de Quito University, Quito, Ecuador.

### 2.3 DNA extraction

Total DNA was extracted from individuals of engorged and unengorged *C. insignis* females, with a modified protocol based on metal chelating agent Chelex-100 resin (BioRad) (Casquet et al., 2012). A volume of 100 μl of Chelex 10% was added to each sample, homogenized with a sterile pestle, centrifuged for 15 s at 2000× *g*, incubated for 30 min at 56 °C with 5 μl of proteinase K, vortexed and then incubated for 30 min at 95 °C and centrifuged for 15 s at 2000× *g*. The supernatant was transferred to a 1.5-ml Eppendorf tube and stored at −20 °C.

### 2.4 Molecular detection of BTV

BTV was detected using a RT-qPCR protocol for the pan-detection of BTV serotypes that targets segment 9 (Maan et al., 2015). A synthetic 127 bp ssDNA molecule corresponding to segment 9 of BTV (Macrogen, Korea) was used as a positive control.

### 2.5 Molecular identification of blood meals

The molecular identification of blood meals was performed using the protocol described by Hadj-Henni et al. (2015) for markers *cyt*b and PNOC. A no-template control was included in each run. The PCR products were sequenced at Macrogen (Korea) and were compared to homologous sequences in GenBank using the nucleotide-nucleotide Basic Local Alignment Search Tool (BLAST). Sequences were aligned with BioEdit v.7.0.0 software for comparison (Hall, 1999). A sequence homology of ≥ 98% was used for species determination (Ninio et al., 2011).

## 3 Results

### 3.1 *Culicoides* spp. and *C. insignis* identification

**Fig. 2:**
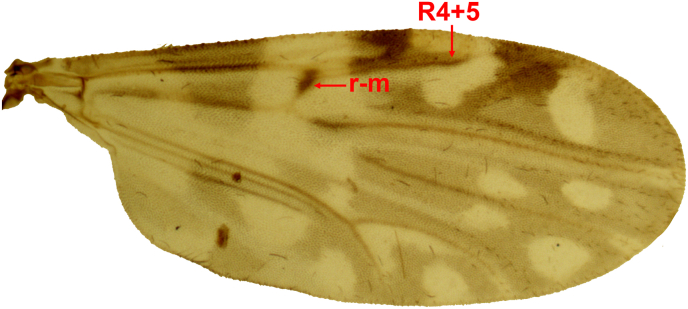
Wing from a *Culicoides insignis* specimen collected in Cotundo. The dark r-m cross-vein and the dark R4+5 vein that turns forward towards the costa are distinctive features of this species (Spinelli et al., 1993).

A total of 1773 female *Culicoides* specimens were identified, of which 326 (18.38%) were *C. insignis* (Table 1, Fig. 2). The proportion of *C. insignis* females was higher in Cotundo (26.50%) compared to Paraíso Escondido (2.65%). Sampling was conducted at 20 different points in Cotundo and 11 points in Paraíso Escondido, at approximately 700 m a.s.l. for all sites. The majority of the remaining *Culicoides* specimens belonged to other species within the subgenus *Hoffmania* based on their wing patterns.

**Table 1:** Number of *Culicoides* spp. specimens collected by locality and RT-qPCR results.

Locality	Female (*C. insignis*)	Engorged *C. insignis*	RT-qPCR BTV-positive
Cotundo	1170 (310)	6	1
Paraiso Escondido	603 (16)	0	0

### 3.2 Molecular detection of BTV

Out of the 304 unengorged *C. insignis* females collected in Cotundo (latitude: 0.8506°, longitude: 77.79498°), only one specimen collected in April 2017 tested positive for BTV (tested in duplicate, mean Ct-value: 23.05, Table 1). This corresponds to an estimated infection rate of 0.33% (95% CI: 0.06–1.84%) in the *C. insignis* population from Cotundo. The presence of BTV was confirmed by sequencing. No unengorged *C. insignis* females were positive for BTV in Paraiso Escondido.

### 3.3 Blood meal analysis

The analysis was successful in four out of six engorged *C. insignis* females from Cotundo and identified horses (*Equus caballus*) and humans (*Homo sapiens*) as their blood meal sources. Among these four specimens, *cyt*b was successfully amplified for three (GenBank: PV111753, PV111754, and PV111755), while PNOC was amplified for all four (GenBank: PV111756, PV111757, PV111758, and PV111759). All positive samples for the blood meal analysis were collected in April 2017 (latitude/longitude: 0.85027°/–77.79557°, −0.85053°/–77.79520°, and −0.85060°/–77.79500°). No engorged *C. insignis* females were found in Paraiso Escondido. It is worth noting that, although both sampling sites at Paraiso Escondido and Cotundo are located on cattle farms, no cattle were present at either site during the collection period, as the animals had been moved to other pastures without prior notice.

## 4 Discussion

To our knowledge, this is the first study to confirm the presence of BTV RNA in *C. insignis* in Ecuador, which supports the incrimination of this species as a vector of BTV in the country. The high viral load detected (Ct-value of 23.05) strengthens this possibility, as values below 25 have been associated with potential virus replication in female *Culicoides* spp. (Foxi et al., 2016). However, RT-qPCR results must be interpreted with caution, as no distinction can be made between viral RNA from the digestive tract (non-infectious) or the salivary glands (infectious) of the insect (Mellor et al., 2000).

The estimated BTV infection rate in *C. insignis* in this study (0.33%) is within the lower range reported for other field-collected *Culicoides* species (Tabachnick, 1996; Goffredo et al., 2015; Foxi et al., 2016). Nonetheless, the wide confidence interval indicates the need for a larger sample size to improve statistical confidence and strengthen epidemiological inference. Infection rates can also vary widely depending on geographical location, collection period, virus circulation in vertebrate hosts, and the diagnostic methods used (Tabachnick, 1996; De Liberato et al., 2005; Savini et al., 2005; Mehlhorn et al., 2007; Dijkstra et al., 2008; Vanbinst et al., 2009; Goffredo et al., 2015; Foxi et al., 2016; Daif et al., 2024).

In Cotundo, where the BTV-positive midge was found, *C. insignis* represented over 25% of all *Culicoides* specimens captured, which reinforces its potential role as BTV vector in Ecuador, as the presence of both the virus and the suspected vector is a key criterion for considering an arthropod as a potential biological vector (Standfast and Dyce, 1972; DeFoliart et al., 1987). This finding is consistent with the established vector status of *C. insignis* in Central America and the Caribbean (Legisa et al., 2014). In contrast, *C. insignis* accounted for only 2.6% of the *Culicoides* captured in Paraíso Escondido, where no engorged or BTV-positive *C. insignis* females were found. The limited geographical coverage, number of sampling points, and the low number of collected specimens may have underestimated the true presence of *C. insignis* and BTV circulation in both localities. Variations in host availability across space and time can also influence the presence and feeding preferences of *Culicoides* species (Lyimo and Ferguson, 2009; Zimmer et al., 2015).

Cattle population number is associated with a higher probability of BTV occurrence in transition zones between the Amazon and Highland regions of Ecuador (Acosta et al., 2025). Pichincha Province (Paraíso Escondido) has one of the largest cattle populations in the country (375,447 animals), compared to 49,519 in Napo (Cotundo) (Acosta et al., 2025). However, the absence of cattle at the time of sampling, due to their relocation to other pastures without prior notice, likely influenced both the vector population dynamics and the results of the blood meal analysis. Although *C. insignis* is known to prefer cattle, it also feeds on other vertebrates, such as horses and humans (Sloyer et al., 2023). In our study, two of the four engorged *C. insignis* females (found only in Cotundo) fed on horses, which were only observed near traps in Cotundo and are not considered BTV reservoir hosts. Although no ruminants were identified as blood meal sources, BTV infection in *Culicoides* may persist for the insect’s lifetime (Mellor, 1990), allowing for viral detection even in the absence of currently infected vertebrate hosts.

Overall, while our findings provide initial molecular evidence of BTV presence in a potential vector species, broader geographical sampling and higher specimen numbers are necessary to improve our understanding of the epidemiological role of *C. insignis* as a BTV vector in Ecuador.

## 5 Conclusion

The detection of BTV RNA in a field-collected, unengorged specimen signals the importance of entomological surveillance for understanding the epidemiology of BTV and related arboviruses. Blood meal analysis indicates that *C. insignis* feeding behavior is likely influenced by host availability. Strengthening vector surveillance and integrating molecular screening into national animal health programmes could help prevent future outbreaks. Future studies should be aimed at expanding both the geographical coverage and sample size to improve the identification of *Culicoides* species involved in BTV transmission, especially in areas where virus circulation in cattle has been documented.

## CRediT authorship contribution statement

**Juan D. Mosquera:** Conceptualization, Methodology, Validation, Formal analysis, Investigation, Data curation, Writing – original draft, Writing – review & editing, Visualization. **Sonia Zapata:** Conceptualization, Methodology, Resources, Funding acquisition, Writing – review & editing, Supervision. **Denis Augot:** Conceptualization, Methodology, Resources, Funding acquisition, Writing – review & editing, Supervision.

## Ethical approval

Not applicable.

## Data availability

The data supporting the conclusions of this article are included within the article. The newly generated sequences for the blood meal sources were submitted to the GenBank database under the accession numbers: PV111753, PV111754, PV111755, PV111756, PV111757, PV111758, and PV111759.

## Funding

This study was supported by a Collaboration Grant from the Universidad San Francisco de Quito (USFQ), Quito, Ecuador.

## Declaration of competing interests

The authors declare that they have no known competing financial interests or personal relationships that could have appeared to influence the work reported in this paper.

## Data Availability

The data supporting the conclusions of this article are included within the article. The newly generated sequences for the blood meal sources were submitted to the GenBank database under the accession numbers: PV111753, PV111754, PV111755, PV111756, PV111757, PV111758, and PV111759.
